# Effect of juggling therapy on anxiety disorders in female patients

**DOI:** 10.1186/1751-0759-1-10

**Published:** 2007-05-01

**Authors:** Toshihiro Nakahara, Kazuhiko Nakahara, Miho Uehara, Ken-ichiro Koyama, Kouha Li, Toshiro Harada, Daisuke Yasuhara, Hikaru Taguchi, Sinya Kojima, Ken-ichiro Sagiyama, Akio Inui

**Affiliations:** 1 Department of Behavioral Medicine, Kagoshima University Graduate School of Medical and Dental Sciences, and Department Psychosomatic Medicine, Respiratory and Stress care Center, Kagoshima University Hospital, Kagoshima City, Japan; 2 Health Art Clinic Kumamoto, Kumamoto City, Japan

## Abstract

**Aims:**

The aim of this study was to investigate the effect of juggling therapy for anxiety disorder patients.

**Design and Method:**

Subjects were 17 female outpatients who met the DSM-IV diagnostic criteria for anxiety disorders. Subjects were treated with standard psychotherapy, medication and counseling for 6 months. For the last 3 months of treatment, subjects were randomized into either a non-juggling group (n = 9) or a juggling therapy group (juggling group: n = 8). The juggling group gradually acquired juggling skills by practicing juggling beanbags (*otedama *in Japan) with both hands. The therapeutic effect was evaluated using scores of psychological testing (STAI: State and Trate Anxiety Inventry, POMS: Profile of Mood Status) and of ADL (FAI: Franchay Activity Index) collected before treatment, 3 months after treatment (before juggling therapy), and at the end of both treatments.

**Results:**

After 6 months, an analysis of variance revealed that scores on the state anxiety, trait anxiety subscales of STAI and tension-anxiety (T-A) score of POMS were significantly lower in the juggling group than in the non-juggling group (p < 0.01). Depression, anger-hostility scores of POMS were improved more than non-jugglers. In the juggling group, activity scores on the vigor subscale of POMS and FAI score were significantly higher than those in the non juggling group (p < 0.01). Other mood scores of POMS did not differ between the two groups.

**Conclusion:**

These findings suggest that juggling therapy may be effective for the treatment of anxiety disorders.

## Findings

Several therapies, including anxiolytics, psychotherapy, and cognitive behavioral therapy (CBT), are available for the treatment of anxiety disorders. Although little research on alternative therapy was conducted in the past, several recent studies have reported their efficacy in patients with posttraumatic stress disorder (PTSD) [1], general anxiety disorders (GAD) through herbal therapy [2], anxiety and mood disorders through several complementary therapies [3], anxiety disorders through yoga therapy [4], and anxiety disorders through meditation and relaxation [5].

Eye movement desensitization and reprocessing (EMDR) is an integrative psychotherapy approach that has been consistently evaluated as effective for treating several anxiety disorders, inclucing PTSD [6], panic disorders [7], and phobias [8]. Although conflicting data has been reported for the efficacy of EMDR [9], this therapy is considered to be of low to moderate level of efficacy [10]. Originally, research on this therapy found that moving the eyes rapidly in a side-to-side motion reduced disturbing thoughts and related anxiety [11]. Currently rapid eye movement methods are occasionally replaced by smooth pursuit eye movement and bilateral stimulation. Smooth pursuit eye movement, bilateral tapping and bilateral tones have been found to be as effective as rapid eye movement [12].

Juggling (*otedama*) has a 3000-year history in Jaoan [13], first appearing in the Nara and Heian Periods (8^th ^– 9^th ^centuries). To date, such games have continued to grow in popularity. A previous report has indicated that three-ball cascade juggling facilitates the growth of gray matter in the mid-temporal lobe [14]. And a previous report suggested that mid-temporal lobe structures may relate to explicit conditioning tasks [15]. Physical movement via meditation [5], and yoga therapy [4] may reduce anxiety through relaxation. With regards to anxiety disorders, a report suggested the involvement of the temporal lobe in the generation of panic attack [16]. This finding suggests that visual motion information and physical movement might improve the psychoneurogical network.

Herein, we present the first trial to investigate the therapeutic effect of juggling on anxiety disorders. We hypothesize that juggling therapy contributes to improvement in patient anxiety through changes in emotional memory processing.  

Subjects in this study were 17 female outpatients with anxiety disorders who met the DSM-IV diagnostic criteria for 6 panic disorder (PD), 4 PTSD, 4 obsessive-compulsive disorders (OCD), and 3 GAD. No subjects had substance use, alcohol or other comorbidities. All subjects were treated with standard psychotherapy, medication and counseling. During the 6-month study period, anxiolytics and antidepressants were prescribed; however, the doses were not changed during the study. For the last 3 months of treatment, subjects were randomized into a non-juggling group (n = 9: 3 PD, 2 PTSD, 2 OCD, and 2 GAD) or a juggling therapy group (juggling group; n = 8, 3 PD, 2 PTSD, 2 OCD, and 1 GAD). Individuals in the juggling group were taught classic beanbag juggling and gradually acquired juggling skills by practicing juggling beanbags with both hands (subjects started with 2 bags, then progressed to 3 bags). They routinely exercised about 5 minutes, twice a day.

The difference in the therapeutic effect was estimated using scores on the State-Trait Anxiety Inventory (STAI), Profile of Mood Status (POMS), and Franchay Activity Index (FAI), collected before treatment, after 3 months of treatment (before juggling therapy), and at the end of both treatments (6 months).

Regarding statistical analysis, psychological test scores were compared using repeated measurement of ANOVA and post-hoc Scheffe's test. All results were considered significant at p < 0.05.

In accordance with the principle of the Declaration of Helsinki, we obtained the written informed consent from participants before enrollment.  

No differences between the 2 groups were observed in any demographic characteristics. Mean ± SD age was 32.7 ± 3.2 years in the non-juggling group and 38.5 ± 3.5 in the juggling group. Duration of disease period was 18.4 ± 6.5 months and 17.0 ± 7.8 months, respectively (mean ± SD).

**Figure 1:**
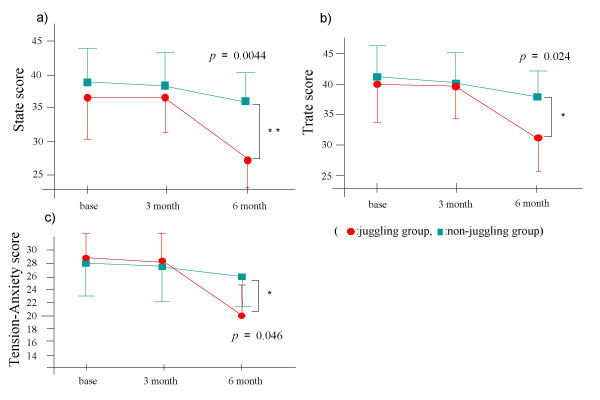
**Change of anxiety scores (mean ± SD)**.  **a)**  State of STAI, **b)** Trate of STAI, ** c)** T-A (Tension-Anxiety) score of POMS, * p < 0.05  and ** p < 0.01 vs. controls.

Regarding anxiety scores (state and trait subscales of the STAI, T-A (tension-anxiety) score of POMS), repeated measurement of ANOVA showed for the group (p = 0.12 and F(1,15) = 2.81, p = 0.32 and F(1,15) = 1.08, p = 0.53 and F(1,14) = 0.33, respectively), time course (p < 0.0001 and F(2,30) = 16.67, p = 0.0002 and F(2,30) = 11.8, p = 0.01 and F(2,28) = 6.23, respectively), and time course × group (p = 0.011 and F(2,30) = 5.37, p = 0.021 and F(2,30) = 5.07, p < 0.0001 and F(2,28) = 17.26, respectively). Anxiety scores in the juggling group were significantly lower than in the non-juggling group at the end of treatment (p = 0.0044, p = 0.024, p = 0.046, respectively) (Figure 1a,b,c).

According to the activity scores (vigor score of POMS and FAI score), repeated measurement of ANOVA showed for the group (p = 0.75 and F(1.15) = 0.10, p = 0.69 and F(1,15) = 0.17, respectively), time course (p < 0.0001 and F(2,30) = 58.40, p < 0.0001 and F(2,30) = 31.82, respectively), and time course × group (p < 0.0001 and F(2,30) = 23.08, p = 0.0011 and F(2,30) = 8.65, respectively). The activity scores of the juggling group were significantly improved in comparison with the non-juggling group.

Scores on the depression, anger-hostility, subscales of POMS, repeated measurement of ANOVA showed for the group (p = 0.33 and F(1,15) = 1.07, p = 0.53 and F(1,15) = 0.41, respectively), time course (p < 0.0001 and F(2,30) = 72.86, p < 0.0001 and F(2,30) = 24.76, respectively), and time course × group (p < 0.0001 and F(2,30) = 40.26, p = 0.023 and F(2,30) = 4.27, respectively). Depression and anger-hostility scores were significantly lower in the juggling group than in the non-juggling group at the end of the treatment (p = 0.0074, p = 0.026, respectively).

In contrast, scores on the confusion and fatigue subscales of the POMS, repeated measurement of ANOVA showed for the group (p = 0.31 and F(1,15) = 1.11, p = 0.79 and F(1,15) = 0.07, respectively), time course (p < 0.0001 and F(2,30) = 33.67, p < 0.0001 and F(2,30) = 94.84, respectively), and time course × group (p = 0.80 and F(1,15) = 0.22, p = 0.80 and F(1,15) = 0.23, respectively). At the end point these scores did not significantly differ between the groups (p = 0.213, p = 0.813, respectively).  

In the present study, we found that anxiety scores in the juggling group were reduced more significantly than those in the non-juggling group. This finding suggested that juggling therapy may reduce anxiety through a visual motion information processing network such as EMDR. Eye movement was reported to reduce anxiety-causing memories or reduce the vividness of such memories [17], and faster resolution may have been obtained with regards to anxiety and emotional distress in the juggling group than in the non-juggling group. The activity of the ACC and lateral prefrontal cortex (PFC) is changed in individuals with higher anxiety levels [18], and the dorsal region of ACC is related to interoceptive awareness [19]. Thus, improvement on anxiety scores through juggling may have resulted from such changes in emotional memory processing and local brain activation [20].

On the other hand, juggling therapy or similar body work therapy may have facilitated changes in the patient's condition through relaxation. Body work therapy like yoga [4], meditation and relaxation [5] are reported to be effective in emotional control. Disrupted attentional control over the threat of anxiety was reported [21], therefore the body sensation associated with juggling therapy may have helped attenional focus control and assisted the homeostatic process such as EMDR [20]. Since mood scores such as confusion and fatigue were not improved, juggling therapy may have a specific and limited influence on the activity of the brain, among other alternative therapies.

Several limitations of this study must be identified. Firstly, because the number of participants was small, we used a broad definition of anxiety disorders. But a report indicated that anxiety disorder may have overlapping pathologies [22]. Secondly, as the therapeutic effect was estimated using only psychological testing without assessing brain function. Hypo- rather than hyper-activation of the PFC has been reported in PTSD patients during the verbal fluency test [23]. Therefore, further research using NIRS, fMRI, PET, and/or SPECT is required to examine the brain sites responsible for the therapeutic effect of juggling.

In conclusion, we demonstrated the anxiolytic effect of juggling therapy in patients with anxiety disorders. Juggling therapy could be performed easily in combination with other forms of therapy for patients with increased anxiety levels.

## Authors' contributions

KN gave the idea of this study. TH, DY, SK and KS helped to collect participant data. DY, MU, KK, KL and HT helped estimate and analyze the data. AI conceived the study design and coordinated and drafted this manuscript. All authors read and approved the final manuscript.
